# Monthly Summary

**Published:** 1896-09

**Authors:** 


### Monthly Summary.

MEDICAL HINTS.—Selected from a contribution by Chas. H. Merz, A. M., M. D., Sandusky, Ohio, in "Columbus Medical Journal" :—

Let the wise physician remember that a patient is often easier lost than gained.

A little common sense often helps a doctor more than all his book-learning and theorizing.

To employ a babbling physician is to gain a worse malady than the one we have.—Meander.

It is one thing for a doctor to understand his business. It is another to understand how to attend to it.

Stupidity is easily pardonable in a doctor, but it is difficult to forgive meanness of heart, purpose and action.

The Talmud says that fruit must grow slowly and ripen of itself. The same is true of the medical student.

The good doctor is cheap at any price, for the same reason that a poor doctor is dear no matter how small his compensation.

The best evidence of higher medical education is the lengthening of the time of study and the recitation plan of teaching.

Be watchful of the doctors that have so much to say about their reputations. Physicians should let others sing their praises. Unfortunately in these days, self-praise often goes a great ways towards securing success.

Confidence is as powerful an agent as skill. Without it the physician strives in vain for success. People consult only the physician in whom they have confidence. Without the trust of your patients, your work is always in vain.

Some plants grow upon the nourishment taken from others; so some physicians live and thrive upon the practice of others. They are medical parasites. The man of worth strikes his own roots, spreads his own branches and bears his own fruit.

The honest doctor has only his patient's good in view, not his own. He labors for mankind, not to stuff his owu pocketbook. He demands pay not because it enriches him, but because he must live that he may be enabled to continue his good work.

The public does not like a croaker—a constant fault-finder. The doctor that occupies his patient's time telling them what a hard and thankless task his is, is in danger of losing his patient. The patient usually comes to the doctor to tell the same story.

When attending society meetings one often meets the dogmatic physician, who is not a pleasant person to associate with. He knows he is right, and that settles the question. He does not care to listen, but would have every one give ear to what he has to say.

It is better to be true to one's self, to be dignified, to pursue the even tenor of one's way, no matter how much others attempt to belittle one. The weak man stops to listen what the people on the street have to say; the man of dignity and purpose has neither time nor inclination to do so.—*Medical World.*

---

THE LIFE AND GROWTH OF THE TOOTH.—The full apprehension of the fact that the different formations which make up the body of the tooth receive life and nourishment from two distinct sources, the pulp and the pericementum, and of the phenomena arising from it, will greatly assist in establishing rational and substantial methods for the treatment and saving of teeth. The pulp, which is made up of nerve-tissue and of vessels for the supply of nutriment and for taking up waste, is the important factor of every tooth. To it is committed the care of the newly erupted tooth, to readjust, recalcify, consolidate, strengthen and sustain the enamel and dentine. Hence the importance of keeping the pulps of young teeth in a condition of healthful activity, carefully guarding them against any encroachment through decay or manipulation. The teeth in mastication, for children, is of more importance than inheritance as a factor in determining the character of the teeth. Natural and healthful mastication, the true cleaner of the teeth, would alone force that exercise required by the pulp and peridentum. Devitalization of the pulp at an early period of life carries with it retrogressive changes in the quality of tooth-material without power to arrest it.

So general is the action of the pulp in rearranging, depositing and solidifying the materials of the dentine and enamel of young teeth, that a tooth, fragile and imperfect at eruption, if introduced into conditions of use and cleanliness, and the pulp protected, may be built into an organ of good type, practically decay-resisting. Isolated instances, of this have been observed, but are regarded as exceptional rather than as the true expression of pulp action. Are not the teachings of the past and present wanting in giving the position of supreme importance to operations on the externals of the tooth, and would it not be more in harmony with the true science of tooth-building to emphasize pulp-protection in young life?—*Cosmos.*

---

Three months ago I had a patient with a lower second molar with a large crown cavity lingual surface. It was almost broken away to the gum line, and a large opening through the bifurcation, where the gum was exposed in cavity. I removed all decay, then used a piece of 22k. gold, 28 gage, and cut a piece to cover bottom of cavity. Then placing gold on a piece of lead, and taking a large shot, I drove it down to make a depression deep enough to have bearing only on the sides of the cavity and none on the gum in the center of cavity, fastening gold disk in place with cement to keep it from moving while packing in amalgam. It is doing well.—C. D. Hertz, in *Items of Interest.*

---

A LOCAL OBTUNDENT.—By placing in the nares a little pledget of cotton caturated in a ten per cent solution of cocain, leaving it for ten minutes, an upper central or lateral incisor may be so benumbed that it can be extracted or excavated with almost no pain. The nerve is anesthetized before it enters the root of the tooth.—Dr. Dawbarn, in *International.*

METHOD OF VERIFYING THE QUALITY OF ALCOHOL.—According to *Cosmos*, Dr. Coiffier has recently made known a very simple process of quickly verifying the quality of alcohol. The process consists simply in igniting in a saucer 20 grains of the alcohol to be tested and in attentively examining the different phenomena that occur during the combustion. The purest alcohol burns with a uniform blue flame without smoke, disengaging an agreeable odor, and without leaving any residue. Now, there is none of the substances used for sophisticating alcohol that does not modify the method of combustion of the latter. Thus the inferior alcohols, the ethers, the fatty acids, all oleaginous substances, essence of turpentine, benzin, etc., even in extremely minute quantity, cause the appearance in the blue flame of long white or yellow fugacious trains of light that stand out clearly from the blue ground of the flame. The presence of foreign substances in alcohol also renders the flame of the latter smoky, as may easily be seen by holding a cold saucer over it. If the alcohol is supercharged with foreign substances, the saucer will become covered with a more or less abundant carbonaceous deposit.—*Items of Interest.*

---

HOW TO DISGUISE THE TASTE OF COD LIVER OIL.—To disguise the taste of cod liver oil is an advantage which may be obtained by the following plan :

Four hundred grammes of oil are mixed with 20 of freshly roasted and ground coffee and 10 of animal charcoal. The whole is kept in a water bath at 140 degrees F. for 15 minutes in a stoppered flask. It is shaken occasionally for two or three days and then filtered through paper. The oil is limpid and light colored, and tastes and smells strongly of coffee.—*Times and Register.*

---

FOR SEPARATING TEETH.—I use a little piece of sea-tangle tent, wedged tightly in the cavity or between the teeth. It is much nicer than rubber and does not produce nearly so much soreness of the teeth.—H. H. Gantz, in *Items of Interest.*

CUSPID ANGLES.—I have observed more failures (recurrences) at cusp angles than at any other point.

This is caused largely by the former practice of leaving these angles intact, with the view of giving some support to the filling, which I believe to be a fallacy. When but little dentine is left it is entirely inadequate to sustain the easily cleaved enamel under the wear and tear of mastication.

Anchorage gives better access to the cavity margins, and all parts of the cavity likewise, than the older forms.

Outward bevel all around the cavity margin (not too great) and the cavity is ready for the filling.

We go to our meetings and we talk and talk about how to prepare cavities and introduce fillings to make them last. Now what more is there to it than the putting of all the material you can in a good, clean, properly prepared hole?

However simple the general principles may seem, experience teaches there is a wide difference as to how the hole is cleaned and the material is put in—whether the results are to be permanent or temporary, comfortable or painful——whether put in by the master or by the apprentice, the accomplished operator or the bungler.

The cervical margin! Place your filling material evenly and firmly on all margins and surfaces alike, maintain as uniform density as possible through the whole mass of the filling. Restore the lost form of the tooth caused by decay and excavation. The cervical margin should now be able to take care of itself.

Amalgam is to take rank, on its merits, as a permanent filling material, both for rich and poor; for resisting decay; for easier form-building, and for its adaptability to the cervical margin.—*Review.*

---

A REMEDY FOR THIRST.—Thirst and great dryness of the mouth in sickness is often relieved by a teaspoonful of powdered gum arabic, beaten thoroughly with a couple of teaspoonfuls of glycerin, to which is added a glass of cold water and enough lemon juice to make the mixture palatable. the mixture may be taken freely, with great relief to the dryness of the mouth and thirst.—*Medical Times.*

BACTERIOLOGISTS.—These are useful assistants, but they are tyrannical masters, and the results of a given treatment must, after all, be judged, not in the laboratory, but in the hospital ward and the sick-room. A check must be imposed on garrulous bacteriologists who show a disposition to ride the cock-horse among us. We are grateful to them for such assistance as it may be in their power th render to medical science, but we cannot allow them to dictate to us what conclusions we are to draw from clinical investigation. Bacteriological statements are of inference, but clinical observations are facts; facts, too, which concern us more nearly than the interesting, but too often contradictory, deductions which foreign laboratory men foist on us at the point of the scalpel.—*Medical Record.*

---

WITH A POISONED LANCET.—At Covington, Ga., on February 26th, Dr. J. C. Anderson, the well-known physician and surgeon who lives at Anderson's station, on the Middle Georgia and Atlantic railroad, six miles below here, came to covington and opened an abscess in a horse's shoulder.

In the course of an operation the doctor cut his hand. On Monday his hand became badly swollen, which showed conclusively that the poison had entered his system.

Since that time he has had a high fever, and has suffered a great deal. He has been receiving the most careful attention, but his condition is now looked upon as very serious.

The doctor has many friends who sincerely sympathize with him in his sad misfortune and would be glad to see him rapidly restored to health.—*Constitution.*

---

FOR SENSITIVE DENTINE, nitrate of silver, we think easily holds superiority. In its use, we formerly applied the remedy at one sitting, making appointment the next day for operating. Experience proves that a wait of half an hour after applying the caustic is better than the longer time. We have found that the most satisfactory form in which to use it, is a saturated solution; the prepared pellets of various kinds soon lose their effectiveness.—*Western Journal.*

ROOT STERILIZATION.—Dr. J. Y. Crawford says on the subject of root sterilization: Isolate the tooth with rubber-dam. Dry the cavity as much as possible before removing foreign contents. During the entire operation sterilize by frequent use of bichloride of mercury, not stronger than one to three-thousandths. When thoroughly cleansed and perfectly dried, close up for a few minutes with a dry tampon of cotton to allow the last bichloride to act on any bacilli present. Pack the canal with cotton saturated with pure German beechwood creasote, as an antiferment, and for its anodyne effect on the peridental membrane. When this test of asepsis is satisfactory fill as desired.—*Southern Dental Journal.*

---

A HALF CENTURY MARK IN DENTAL EDUCATION.—The semi-centennial anniversary of the Ohio College of Dental Surgery was appropriately celebrated at Cincinnati, Ohio, April 16th, 1896, by memorial exercises and the erection of a tablet by the alumni in honor of the founder, Dr. James Taylor. Too much praise cannot be given the pioneers in the regions of dental professional education, which in its literature and teaching institutions has won for this country a foremost rank in the domain of dental culture. Every dentist is to-day a debtor to Harris, Taylor, and the other founders and promoters of the original colleges for dental instruction. It is in recognition of this obligation that we direct attention and accord space to the memorial address of Proffessor Cassidy.—*Dental Cosmos.*

---

A SUBSTITUTE FOR GOLD.—A French journal describes a new and promising substitute for gold. It is produced by alloying ninety-four parts of copper with six of antimony, the copper being first melted and the antimony afterward added. To this a quantity of magnesium carbonate is added to increase its specific gravity. The alloy is capable of being drawn out, wrought, and soldered just as gold is, and is said to take and retain as fine a polish as gold. Its cost is a shilling a pound.—*Evening Post*

